# Resveratrol Enhances the Radiosensitivity by Inducing DNA Damage and Antitumor Immunity in a Glioblastoma Rat Model under 3 T MRI Monitoring

**DOI:** 10.1155/2022/9672773

**Published:** 2022-10-13

**Authors:** Liping Qian, Lihua Mao, Weixing Mo, Rong Wang, Yunlong Zhang

**Affiliations:** ^1^Radiology Department, Hangzhou Cancer Hospital, Hangzhou City, 310002 Zhejiang Province, China; ^2^Radiology Department, Yuyao People's Hospital, Yuyao City, 315400 Zhejiang Province, China; ^3^Radiology Department, Hangzhou First People's Hospital, Hangzhou City, 310006 Zhejiang Province, China; ^4^Ultrasonography Department, Linping Campus The Second Affiliated Hospital of Zhejiang University School of Medicine, Hangzhou City, 310005 Zhejiang Province, China

## Abstract

Glioblastoma (GBM) is the most common intracranial tumor with characteristic of malignancy. Resveratrol, a natural originated polyphenolic compound, has been reported to act as a potential radiosensitizer in cancer therapy. Magnetic resonance imaging (MRI) is the first choice for the diagnosis, pathological grading, and efficacy evaluation of GBM. In this study, MRI was applied to observe whether resveratrol could intensify the anti-GBM tumor effect by enhancing antitumor immunity during radiotherapy. We established an intracranial C6 GBM model in SD rats, treated with radiation and resveratrol. The increased body weight, the inhibition on mortality, and tumor volume in radiated- GBM rats were further enhanced by resveratrol addition, while the pathological damage of brain was alleviated. The modulation of radiation on inflammation, cell cycle, and apoptosis was strengthened by resveratrol; and Ki-67, PD-L1, and cell cycle- and apoptosis-related protein expressions were also improved by cotreatment. Besides, cotreatment attenuated DNA damage and induced G0/G1-phase cell arrest of GBM rats, accompanied with the changed expression of ATM-AKT-STAT3 pathway-related proteins. Moreover, the percentages of CD3^+^CD8^+^T cells and IFN-*γ*^+^CD8^+^T cells were enhanced, while (CD4^+^CD25^+^Foxp3)/CD4^+^T cells were decreased by radiation or resveratrol, which was strengthened by cotreatment. The modulation effect of cotreatment on CD3, Foxp3, and IFN-*γ* levels was also stronger than radiation or resveratrol alone. To conclude, resveratrol enhanced the effect of radiotherapy by inducing DNA damage and antitumor immunity in the intracranial C6 GBM.

## 1. Introduction

Glioblastoma (GBM) is the most common intracranial tumor and accounts for more than half of brain tumors [1]. The most significant pathological feature of GBM is the infinite proliferation of tumor cells and high invasiveness [2]. GBM progresses rapidly and can invade into the normal brain tissue, and this invasive growth can lead to gradual increase of intracranial pressure, resulting in weakness of limbs, nausea, dizziness, seizures, and other clinical symptoms [3]. According to the level of invasiveness, GBM can be divided into localized and diffuse types clinically; the former can be treated by total surgical resection, while the latter usually requires adjuvant radiotherapy and chemotherapy after surgery [4]. Despite the standard surgery and chemoradiotherapy, the overall survival of GBM patients remains poor due to the heterogeneity of the tumor tissue and its resistance to treatment [5, 6].

Radiotherapy can ameliorate the symptoms and progression of tumor patients, which has been a critical means for the treatment of GBM [7]. Radiotherapy can damage DNA of tumor cells, achieving the purpose of killing tumor cells [8]. However, tumor cells can actively repair themselves, causing the phenomenon of radiotherapy resistance [9]. What is more, it has been suggested that this resistance may activate the anticancer immunity, which supports the development of tumors [10]. The development and progression of cancer can be influenced by the host's immunological response. During malignancy, cancer cells frequently acquire immunotolerance and aid in the infiltration of immunosuppressive cells in the tumor microenvironment (TME). Therefore, how to further sensitize the effect of radiotherapy and promote the prognosis of GBM patients without increasing side effects is a research hotspot [11].

Traditional Chinese medicine (TCM) has been applied to treat various cancers due to the advantages of targeting multiple signaling pathways, few adverse reactions, and good tolerance [12, 13]. Resveratrol, a polyphenolic compound extracted from natural plants such as grapes, peanuts, and mulberries, can weaken the growth of various human and rat tumors cells both *in vitro* and *in vivo* [14]. Unlike most anticancer drugs, the anticancer dose of resveratrol has no adverse effects on the growth of normal cells and the function of tissues and organs, suggesting that it is safe to be used alone or in combination with other treatments [15]. More importantly, resveratrol can be utilized as a radiosensitizer in the treatment of cancers, including GBM [16]. The combined radiotherapy and resveratrol can effectively induce GBM cell death through multiple pathways by regulation of cell cycle progression, cell proliferation, cell apoptosis, cell autophagy, oxidant system, and so on [17]. In addition, resveratrol is also a promising modulator of tumor immunotherapy [18]. Resveratrol has been reported to manifest the ability to enhance antitumor immunity [19]. Kim et al. have reported that the resveratrol analogue, HS-1793, intensified the function of radiotherapy via inducing antitumor immunity in the growth of breast cancer [20]. However, whether resveratrol enhances the effect of radiotherapy by inducing antitumor immunity and DNA damage during GBM tumor growth in animals has not been investigated clearly.

Magnetic resonance imaging (MRI) has become one of the crucial imaging examination methods for early tumor diagnosis and treatment efficacy monitoring clinically [21]. In this study, we established a rat intracranial GBM model and used a 3-Tesla (T) MRI technology to explore the suppressive effect of resveratrol combined with radiotherapy on GBM growth and the regulation mechanism.

## 2. Materials and Methods

### 2.1. Ethics Statement

#### 2.1.1. Animals

A total of 60 male SD rats, weighing 180-200 g, were acquired from Shanghai SLAC Laboratory Animal Co., Ltd. They were fed adaptively for 7 days and raised in a pathogen-free facility with temperature of 25 ± 2°C, humidity of 55 ± 5%, and 12 h day-night cycle.

### 2.2. Cell Culture

Rat GBM cell line C6 (C3031) was bought from iCell Bioscience Inc (China) and grown in RPMI-1640 medium (31800, Solarbio, China) augmented with 10% FBS. C6 cells were put in a cell incubator (BB150, Thermo Fisher, USA) for culture.

### 2.3. Establishing the Model of Intracranial C6 GBM

We established an intracranial C6 GBM model as previously described [22]. The rats were anaesthetized with 3% isoflurane (792632, Sigma-Aldrich, USA) in an airtight chamber. To keep surgical depth anesthesia during the procedures, anesthesia was maintained by spontaneous respiration of 1% isoflurane. Anesthetized rats were attached to stereoscopic brain locators, and their hair was clipped off the top of their heads. After the surgical area was disinfected, the scalp was cut lengthwise about 1-2 cm to fully expose the surgical area. The fixed rod was adjusted so that the anterior and posterior fontanelles of the rat's skull were on the same plane. We utilized a 1 mm width drill to drill a small hole1 mm anterior and 3 mm lateral to the right bregma. A microsyringe was exploited to withdraw the C6 cell suspension (1 × 10^6^ cells/10 *μ*L), which was then injected into the subdura to a depth of 4 mm. The injection speed was 1 *μ*L/min, and the total injection time was 10 min. After the injection, the needle was retained for 3-5 min and the needle was pulled out slowly. Bone holes were sealed with bone wax to prevent the reflux of cell suspension.

### 2.4. Study Protocol

Rats inoculated with C6 cells for 2 weeks were allocated to 4 groups as follows: normal control group, radiation group, resveratrol group, and radiation+resveratrol group, 15 rats per group. The rats in the resveratrol group and radiation+resveratrol group were given resveratrol orally (40 mg/kg, S30630, Shanghai Yuanye Bio-Technology Co., Ltd, China) once every other day for 14 days. The rats in the radiation group and radiation+resveratrol group were given 5 Gy X-ray irradiation to the head on the 15^th^, 20^th^, and 25^th^ days after inoculation. During the experiment, the general condition, survival rate, and body weight of rats in each group were recorded. The experimental period was 28 days.

### 2.5. MRI Observation of Intracranial C6 GBM Model in Rats

We scanned the rat skull with MRI and calculated tumor volume on days 21 and 28 of the experimental cycle. After anesthesia, 5 randomly selected rats in each group were placed in the prone position and their heads were fixed in a high-resolution scanning loop. Rats were treated with gadolinium contrast agent (G105875, Aladdin, China) by tail vein injection at a dose of 0.1 mL/kg. Animals were imaged under a 3 T MRI scanner (UMR 790, Shanghai Lianying Medical Technology Co., Ltd.). T1WI scan parameters were as follows: field of view (FOV) = 70 × 40 mm, matrix = 256 × 256, section thickness = 2 mm, space = 10 m, and repetition time (TR)/echo time (TE) = 350 milliseconds/24 ms. T2WI scan parameters were as follows: FOV = 50 × 50 mm, matrix = 256 × 256, section thickness = 2 mm, space = 10 mm, and TR/TE = 3964 ms/87.6 ms. GBM volume (*V*) after enhanced MRI was calculated: the maximum transverse diameter (*W*), long diameter (*L*), and high diameter (*H*) of the tumor were measured, *V* = (*W* × *L* × *H* × *π* × 4/3) × 1/8.

### 2.6. Sample Collection

On day 29, rats were subjected to euthanasia by CO_2_. We exposed the heart of rats and obtained blood samples from the right ventricle utilizing heart puncture. The blood samples were centrifuged (4°C, 3000 rpm, 15 min). The upper serum was retained at -80°C. Then, we quickly harvested the brain GBM tissues and spleen tissues. Part of the GBM tissues was immersed in 4% paraformaldehyde (PFA, G1101) provided by Servicebio (China). After being dehydrated and wax-embedded, the tissues were sectioned (4 *μ*m). The remaining GBM tissues and spleen tissues were kept at -80°C.

### 2.7. ELISA

Rat interferon-gamma (IFN-*γ*) kit (PI510), rat tumor necrosis factor-alpha (TNF-*α*) kit (PT516), rat interleukin 6 (IL-6) kit (PI328), and rat transforming growth factor beta (TGF-*β*) kit (PT878) were bought from Beyotime (China). The contents of IFN-*γ*, TNF-*α*, IL-6, and TGF-*β* in rat serum were assessed in strict accordance with the corresponding instructions of IFN-*γ* kit, TNF-*α* kit, IL-6 kit, and TGF-*β* kit.

### 2.8. Hematoxylin-Eosin Staining (H&E) Staining

After xylol dewaxing, the GBM tumor tissue slices were immersed in hematoxylin solution (C0107, Beyotime, China) for 5 min and then rinsed with distilled water for 1 min. After being differentiated into 75% hydrochloric acid ethanol for 30 seconds, the slices were immersed in 1% eosin solution (C0109, Beyotime, China) for 3 min. After dehydrating, xylene was utilized as transparent. The neutral balsam (D054-1-1, Jiancheng, China) was exploited to seal the slices. In the end, the slices were placed in an optical microscope (Z723975-1EA, Sigma, USA) to observe the tissue integrity.

### 2.9. Terminal Deoxynucleotidyl Transferase dUTP Nick End Labeling (TUNEL) Staining

Proteinase K (ST532) offered by Beyotime (China) was applied to treat the dewaxed and hydrated slices (37°C, 0.5 h). After rinsing, the slices were exposed to TUNEL solution (C1086) offered by Beyotime (China), which were then placed at 37°C for 60 min. A fluorescence microscope (IX70) provided by Olympus (Japan) was exploited to observe the stained slices after adding DAPI (C1005, Beyotime, China).

### 2.10. Immunohistochemistry (IHC) Assay

For antigen repair, the slices were reacted with 0.01 M citrate buffer (95-99°C, 10 min, I012-1-1, Jiancheng, China). After being reacted with 0.1% Triton X-100 (ST797, Beyotime, China) at 37°C for 10 min, the slices were exposed to anti-Ki-67 antibody (1 : 100, abx001706, Abbexa, USA) and anti-PD-L1 antibody (1 : 250, ab205921, Abcam, UK) at 4°C for 12 h. A further 1 h reaction was conducted after the addition of secondary antibody (1 : 10000, ab6721, Abcam, UK). DAB (abs9210, Absin, China) was then exploited to treat the slices. Lastly, the images were captured with the aid of the optical microscope after being mounted with neutral balsam.

### 2.11. Flow Cytometric Assay

Spleen tissue was washed with PBS, and then, erythrocyte lysate was added. After centrifugation, the pelleted cells were resuspended in PBS. A flow tube containing 100 *μ*L of cell suspension was added with FITC Mouse Anti-Rat CD4 antibody (561833, BD Pharmingen, USA), FITC Mouse Anti-Rat CD8 antibody (561965, BD Pharmingen, USA), FITC Mouse Anti-Rat IFN-*γ* antibody (559498, BD Pharmingen, USA), and PE Rat anti-Mouse Foxp3 antibody (566881, BD Pharmingen, USA). After reacting for 0.5 h at 4°C in the dark, the flow tube containing cells was rinsed with PBS and then resuspended in PBS. Lymphocyte subpopulations among splenocytes were assessed by a flow cytometer (PAS III, Partec, Germany).

### 2.12. Western Blot

RIPA buffer (PC104, Epizyme, China) was applied to extract protein from GBM tissues followed by centrifugation. The lysed protein was quantified utilizing BCA kit (ZJ101, Epizyme, China). After electrophoresis (10% SDS-PAGE), the protein was blotted onto nitrocellulose membranes. After being sealed, the membranes were immersed in primary antibodies at 4°C overnight. A further 60 min reaction was done after the addition of secondary antibodies. After being developed with an ECL luminescence reagent (P1000, Applygen, China), the signals were analyzed by a gel imaging system. The primary antibodies of Cyclin D1 (1 : 200, ab16663), C-myc (1 : 1000, ab32072), Bad (1 : 1000, ab32445), p-p53 (1 : 5000, ab33889), ATM (1 : 1000, ab201022), Histone H2A.X (1 : 1000, ab20669), p-Histone H2A.X (1 : 1000, ab81299), phospho-STAT3 (Ser727) (1 : 1000, ab32143), phospho-STAT3 (Tyr705) (1 : 10000, ab267373), STAT3 (1 : 2000, ab68153), and GAPDH (1 : 10000, ab181602) were gained from Abcam (UK). The primary antibodies of p-AKT (1 : 2000, #4060), AKT (1 : 1000, #4685), Cleaved Caspase-9 (1 : 1000, #9507), and p-ATM (1 : 1000, #2851) were obtained from CST (USA). PD-L1 (1 : 1000, abx179111) was bought from Abbexa (USA). Pro-Caspase-9 (1 : 2000, AF6348) was bought from Affinity (USA).

### 2.13. Comet Analysis

The splenic tissue treated with erythrocyte lysate was centrifuged. The centrifuged splenocytes were resuspended in PBS and adjusted to a cell concentration of 1 × 10^6^/mL. 0.5% normal melting point agarose (NMA) was added to the slide until the NMA solidified. Next, 10 *μ*L of cell suspension (104) and 75 *μ*L of 0.7% low melting point agarose (LMA) were mixed well and then dropped onto the above NMA. After solidification of LMA, 0.7% LMA was again dropped into the above gel and solidified at 4°C. The slides were placed in a petri dish, and prechilled lysis buffer was added. After lysis at 4°C for 60-120 min, we washed the slides with PBS. The slides were then subjected to electrophoresis. After that, 0.4 mmol/L Tris-HCl buffer was applied to wash the slides. PI dye was dropped onto each slide and stained in the dark for 10 min. Lastly, the slides were analyzed with the help of the fluorescence microscope.

### 2.14. Cell Cycle Assay

A cell cycle kit (C1052, Beyotime, China) was used based on the manual. The frozen GBM tissues were prepared into a single-cell suspension. After centrifugation, the pelleted cells were subjected to suspension with precooled PBS and shifted onto the centrifuge tube. After centrifugation again, they were fixed with precooled 70% ethanol (4°C, 12 min). After washing, PI staining solution was added. After being reacted at 37°C for 0.5 h, the stained results were assessed with the use of the flow cytometer.

### 2.15. Immunofluorescence Assay

Antigen retrieval was performed in 0.01 M citrate buffer at 95-99°C for 10 min. Next, 5% normal goat serum was exploited to block the slices. Afterwards, the blocked slices were immersed in anti-CD3 antibody (1 : 10, ab135372, Abcam, UK), anti-Foxp3 antibody (1 : 800, #12653, CST, USA), and anti-IFN-*γ* antibody (1 : 100, #8455, CST, USA) at 4°C for 12 h. Goat Anti-Rabbit IgG H&L (Alexa Fluor® 488, ab150077, Abcam, UK) was subsequently performed to treat the slices (37°C, 60 min). In the end, the fluorescence images were obtained by the fluorescence microscope.

### 2.16. Statistical Analysis

Quantitative data were displayed as mean ± standard deviation. All data were representative of triplicate independent experiments, and SPSS software (16.0, IBM, USA) was employed to process data. The multiple comparisons were done with the use of a one-way ANOVA. Comparison between groups was analyzed using the Tukey test. The Kruskal-Wallis *H* test was applied for heterogeneity of variance. *P* < 0.05 implicated the statistical significance.

## 3. Results

### 3.1. The Increase of Body Weight and the Inhibition of Mortality and Tumor Volume in GBM Rats Were Enhanced by Resveratrol

**Figure 1:**
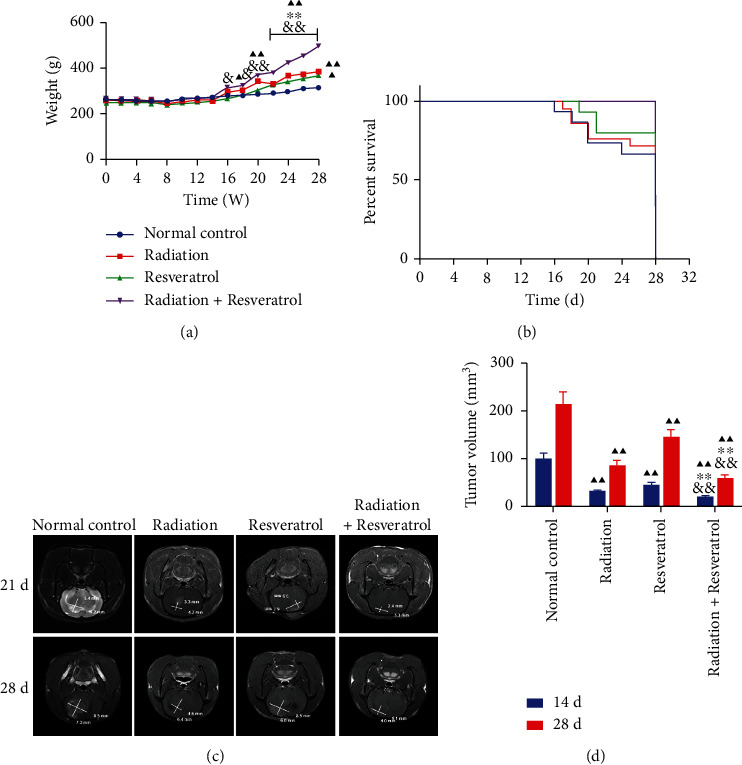
The elevation of body weight and the inhibition of mortality and tumor volume in GBM rats were enhanced by resveratrol. (a) The impacts of radiation and resveratrol on body weight in GBM rats. Data are expressed as mean ± SD, *n* = 8. ^▲^*P* < 0.05 and ^▲▲^*P* < 0.01 vs. normal control; ^∗∗^*P* < 0.01 vs. radiation; ^&^*P* < 0.05 and ^&&^*P* < 0.01 vs. resveratrol. (b) The impacts of radiation and resveratrol on the survival rate in GBM rats, *n* = 8. (c) Image of magnetic resonance imaging (MRI) scanning results. (d) The effects of radiation and resveratrol on tumor volume in GBM rats. Data are expressed as mean ± SD, *n* = 5. ^▲▲^*P* < 0.01 vs. normal control; ^∗∗^*P* < 0.01 vs. radiation; ^&&^*P* < 0.01 vs. resveratrol.

The body weight and mortality of the rats in each group were monitored. The body weight of rats in the radiation+resveratrol group was higher than that in the normal control group on the 24^th^ to 28^th^ day (Figure 1(a), *P* < 0.01), and the body weight of the rats in the resveratrol group enhanced notably on the 28^th^ day (Figure 1(a), *P* < 0.05); the body weight of rats in the cotreatment group was higher compared to that in the radiation or resveratrol group from day 22 to day 28 (Figure 1(a), *P* < 0.05 or *P* < 0.01); the body weight of rats in the cotreatment group enhanced notably than that in the resveratrol group from day 16 to day 28 (Figure 1(a), *P* < 0.05 or *P* < 0.01). Rats in the normal control group died on day 16, while those in the radiation group and resveratrol group died on days 18 and 19, respectively. No rats died in the cotreatment group during the experiment (Figure 1(b)). Then, we scanned the rat skull with MRI and calculated tumor volume on days 21 and 28 of the experimental cycle. The repressive effect of resveratrol and radiation utilized in combination on tumor volume was stronger than that of radiation or resveratrol alone (Figures 1(c) and 1(d), *P* < 0.01).

### 3.2. Cotreatment Alleviated the Pathological Damage of Brain Tissue and Decreased the Expression of Ki-67 and PD-L1

**Figure 2:**
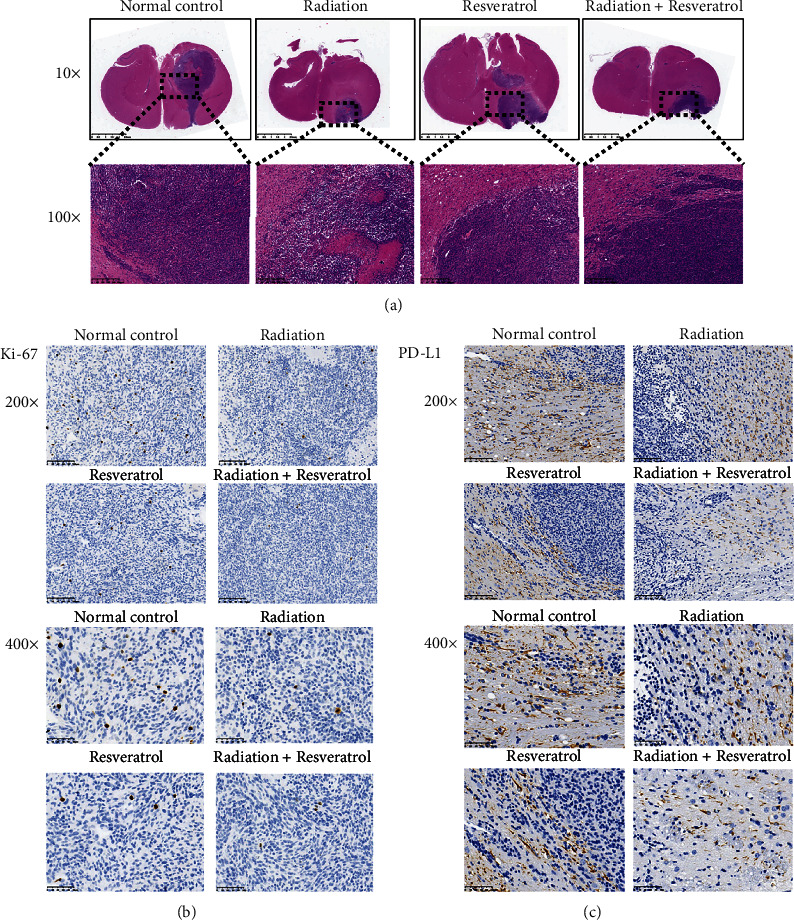
Cotreatment of radiation and resveratrol alleviated the pathological damage of brain tissue and decreased expression of Ki-67 and PD-L1. (a) The impacts of radiation and resveratrol on the pathological damage of brain tissue assessed by H&E staining. Magnification ×10 and ×100, scale bar = 2.5 mm and 200 *μ*m. (b, c) The impacts of radiation and resveratrol on the expression of Ki-67 and PD-L1 assessed by IHC assay. Magnification ×200 and ×400, scale bar = 100 *μ*m and 50 *μ*m.

HE staining showed that the brain tissue of rats in the normal control group was massively infiltrated by the injected GBM cells and formed a complete tumor structure. The brain tissue of rats in the radiation group, resveratrol group, and cotreatment group was also infiltrated with tumor cells to different degrees, but smaller than that in the normal control group (Figure 2(a)). Then, IHC analysis showed that each intervention group impeded the positive expression of Ki-67 and PD-L1, and combined radiation and resveratrol showed stronger suppressive effect (Figures 2(b) and 2(c)).

### 3.3. The Impacts of Radiation and Resveratrol on Inflammation-Related Factors of Serum in GBM Rats

**Figure 3:**
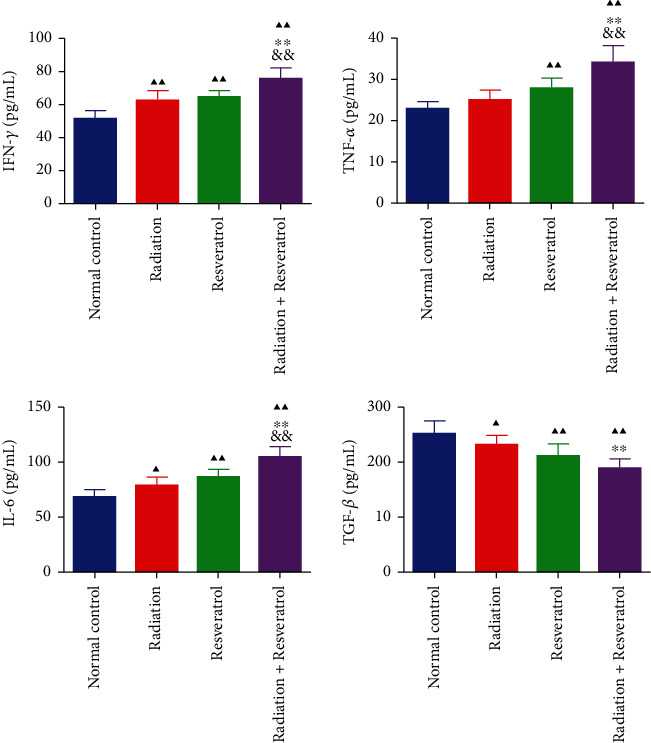
The impacts of radiation and resveratrol on inflammation-related factors of serum in GBM rats. Data are expressed as mean ± SD, *n* = 6. ^▲^*P* < 0.05 and ^▲▲^*P* < 0.01 vs. normal control; ^∗∗^*P* < 0.01 vs. radiation; ^&&^*P* < 0.01 vs. resveratrol.

The contents of IFN-*γ*, TNF-*α*, IL-6, and TGF-*β* in rat serum were assessed. Radiation and resveratrol alone or cotreatment augmented the serum level of IFN-*γ*, TNF-*α*, and IL-6 and decreased the level of TGF-*β* (Figure 3, *P* < 0.01). Cotreatment of radiation and resveratrol had a stronger effect on inflammation-related factors than utilizing resveratrol or radiation alone (Figure 3).

### 3.4. The Effects of Radiation and Resveratrol on Apoptosis and Cell Cycle of Tumor Cells in GBM Rats

**Figure 4:**
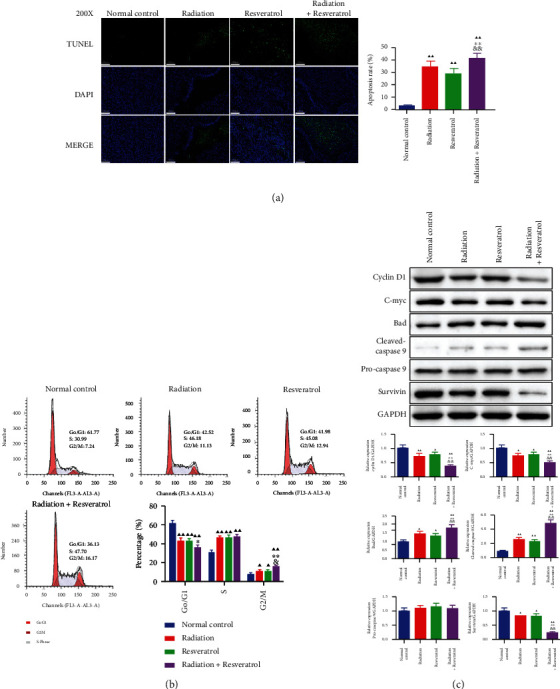
The effects of radiation and resveratrol on apoptosis and cell cycle of tumor tissues in GBM rats (a) TUNEL staining was applied to examine apoptosis of brain tissue. Magnification ×200, scale bar = 100 *μ*m. Data are expressed as mean ± SD, *n* = 6. ^▲▲^*P* < 0.01 vs. normal control; ^∗^*P* < 0.05 vs. radiation; ^&&^*P* < 0.01 vs. resveratrol. (b) The roles of radiation and resveratrol on the distribution of cell cycle were examined by flow cytometry. Data are expressed as mean ± SD, *n* = 3. ^▲^*P* < 0.05 and ^▲▲^*P* < 0.01 vs. normal control; ^&^*P* < 0.05 vs. resveratrol; ^∗∗^*P* < 0.01 vs. radiation. (c) The roles of radiation and resveratrol on cell cycle- and apoptosis-related factors assessed by Western blot. Data are expressed as mean ± SD, *n* = 3. ^▲^*P* < 0.05 and ^▲▲^*P* < 0.01 vs. normal control; ^∗^*P* < 0.05 and ^∗∗^*P* < 0.01 vs. radiation; ^&^*P* < 0.05 and ^&&^*P* < 0.01 vs. resveratrol.

As shown in Figure 4(a), the apoptosis rate of brain GBM tissue in each treatment group was higher than that in the normal control group (*P* < 0.01); the cotreatment group exhibited stronger apoptosis than the resveratrol or radiation group (Figure 4(a), *P* < 0.05). The cell cycle assay showed that the proportion of cells in the G0/G1 phase decreased evidently in all treatment groups and augmented greatly in the S phase and G2/M phase. The combined treatment group had stronger regulation of the proportion of cells in the G0/G1 and G2/M phase than the single intervention group (Figure 4(b), *P* < 0.05). Then, apoptosis- and cell cycle-related markers were assessed (Figure 4(c)). Radiation, resveratrol, or cotreatment effectively suppressed the level of Cyclin D1, C-myc, and Survivin and augmented the expression of Bad and Cleaved Caspase-9 (Figure 4(c), *P* < 0.05). We discovered that combined radiation and resveratrol had a higher modulatory effect on the above-mentioned proteins than utilizing radiation or resveratrol alone (Figure 4(c), *P* < 0.05).

### 3.5. The Combination of Radiation and Resveratrol Attenuated DNA Damage in Splenocytes of GBM Rats

**Figure 5:**
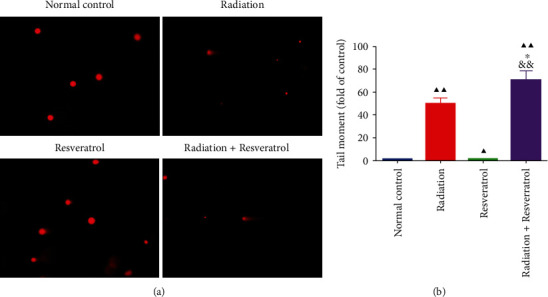
The combination of radiation and resveratrol attenuated DNA damage in splenocytes of GBM rats. (a, b) The roles of radiation and resveratrol on DNA damage in splenocytes of GBM rats assessed by comet analysis. Data are expressed as mean ± SD, *n* = 3. ^▲^*P* < 0.05 and ^▲▲^*P* < 0.01 vs. normal control; ^∗^*P* < 0.05 vs. radiation; ^&&^*P* < 0.01 vs. resveratrol.

**Figure 6:**
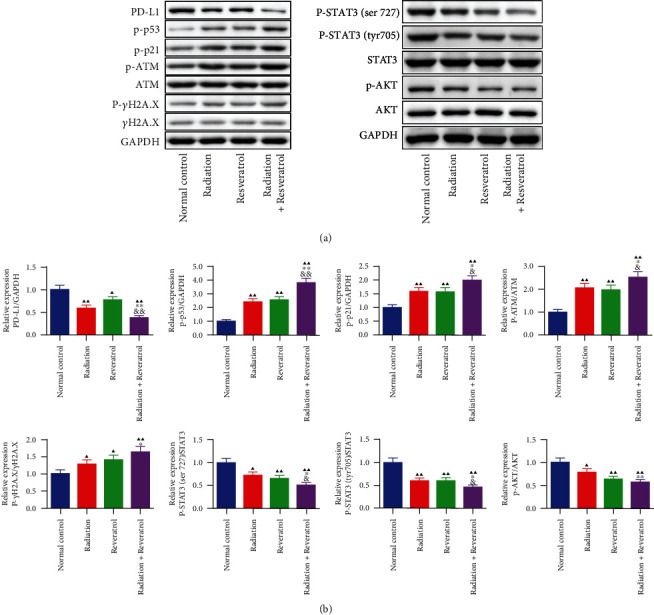
The impacts of radiation and resveratrol on specific proteins of tumor tissues in GBM rats. (a, b) The roles of radiation and resveratrol on ATM-AKT-STAT3-PD-L1 pathway-related factors were assessed by Western blot. Data are expressed as mean ± SD, *n* = 3. ^▲^*P* < 0.05 and ^▲▲^*P* < 0.01 vs. normal control; ^∗^*P* < 0.05 and ^∗∗^*P* < 0.01 vs. radiation; ^&^*P* < 0.05 and ^&&^*P* < 0.01 vs. resveratrol.

The effect of radiation and resveratrol on DNA damage of GBM rats was assessed by the comet analysis. The tail moment of splenocytes in all intervention groups was higher than that in the normal control group (Figures 5(a) and 6(b), *P* < 0.05), and the elevation of cotreatment on the tail moment was higher relative to single treatment (Figures 5(a) and 6(b), *P* < 0.05).

### 3.6. The Impacts of Radiation and Resveratrol on DNA-Damage-Related Proteins in Tumor Tissues of GBM Rats

Thereafter, DNA-damage-related proteins of the ATM-AKT-STAT3-PD-L1 pathway were examined (Figure 6). Radiation, resveratrol, or cotreatment greatly downregulated PD-L1 level as well as the ratios of p-STAT3 (ser 727)/STAT3, p-STAT3 (tyr 705)/STAT3, p-AKT/AKT, and upregulated p-p53 and p-p21 levels as well as the ratios of p-ATM/ATM and p-*γ*H2A.X/*γ*H2A.X (Figures 6(a) and 6(b), *P* < 0.05). Interestingly, combined radiation and resveratrol presented stronger modulatory effect on the expression of these proteins (Figures 6(a) and 6(b), *P* < 0.05).

### 3.7. The Radiation and Resveratrol Cotreatment Modulated the Percentages of T Lymphocyte Subtypes

**Figure 7:**
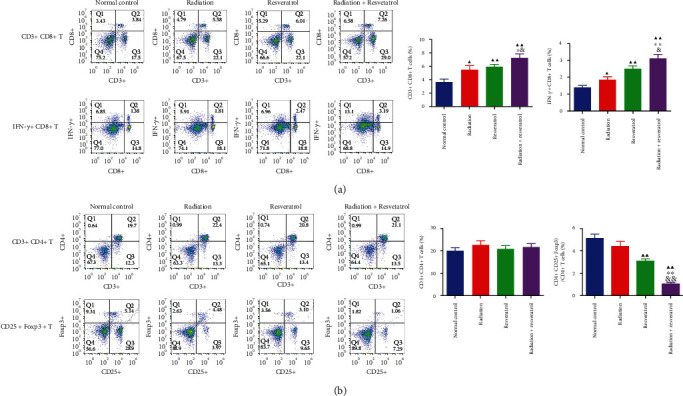
Detection of the percentage of CD3^+^CD8^+^T cells, IFN-*γ*^+^CD8^+^T cells, CD3^+^CD4^+^T cells, and (CD4^+^CD25^+^Foxp3)/CD4^+^T cells in rat splenocytes. (a, b) A flow cytometer was employed to examine the percentage of CD3^+^CD8^+^T cells, IFN-*γ*^+^CD8^+^T cells, CD3^+^CD4^+^T cells, and (CD4^+^CD25^+^Foxp3)/CD4^+^T cells in rat splenocytes. Data are expressed as mean ± SD, *n* = 3. ^▲^*P* < 0.05 and ^▲▲^*P* < 0.01 vs. normal control; ^∗^*P* < 0.05 and ^∗∗^*P* < 0.01 vs. radiation; ^&^*P* < 0.05 and ^&&^*P* < 0.01 vs. resveratrol.

Compared with those of the normal control group, the percentages of CD3^+^CD8^+^T cells and IFN-*γ*^+^CD8^+^T cells were greatly enhanced (Figure 7(a),*P* < 0.05or*P* < 0.01) in treatment groups refer which to the model group, while the percentage of CD3^+^CD4^+^T cells in rat splenocytes in each intervention group had no notable change (Figure 7(b)). The percentage of (CD4^+^CD25^+^Foxp3)/CD4^+^T cells in rat splenocytes in the resveratrol group and cotreatment group was obviously decreased (Figure 7(b), *P* < 0.01). More importantly, the combined intervention of resveratrol and radiation had stronger regulatory effect on the above-mentioned T cells than the single treatment group (Figures 7(a) and 7(b), *P* < 0.05).

### 3.8. The Effects of Radiation and Resveratrol on the Expressions of CD3, CD4, CD8, Foxp3, and IFN-*γ* in Tumor Tissues of GBM Rats

**Figure 8:**
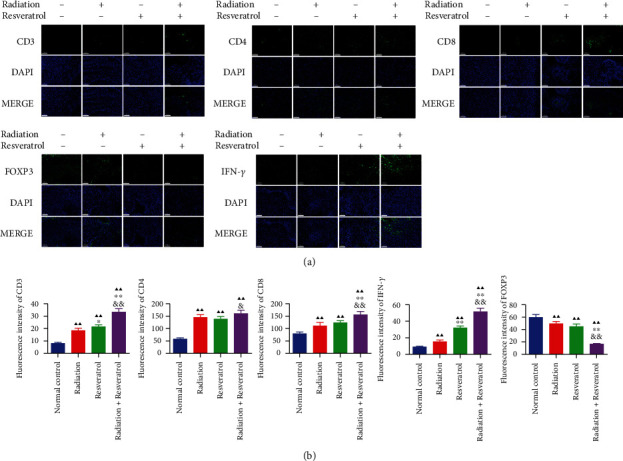
The effects of radiation and resveratrol on the expression of CD3, CD4, CD8, Foxp3, and IFN-*γ* in tumor tissues of GBM rats. (a, b) The role of radiation and resveratrol on the expression of CD3, CD4, CD8, Foxp3, and IFN-*γ* in tumor tissues of GBM rats was assessed by immunofluorescence assay. Data are expressed as mean ± SD, *n* = 6. ^▲▲^*P* < 0.01 vs. normal control; ^&^*P* < 0.05 and ^&&^*P* < 0.01 vs. resveratrol; ^∗^*P* < 0.05 and ^∗∗^*P* < 0.01 vs. radiation.

The immunofluorescence staining illustrated that the positive expression of CD3, CD4, CD8, and IFN-*γ* in GBM tissues was prominently upregulated, while Foxp3 was downregulated by radiation, resveratrol, or cotreatment (Figures 8(a) and 8(b), *P* < 0.01). We further found that the modulation effect of the combined intervention on the expression of CD3, CD4, CD8, Foxp3, and IFN-*γ* was stronger than that of radiation or resveratrol alone (Figures 8(a) and 8(b), *P* < 0.05).

## 4. Discussion

The intracranial C6 GBM cell injection is commonly used to establish an experimental GBM model for basic research as well as for drug target and novel therapy screening [23]. The innovation of this research is that, in the experimental study for the anti-GBM activity of resveratrol combined with radiotherapy, the rat orthotopic GBM model was utilized, the tumor growth was observed by 3 T MRI, and the regulatory effect of combined therapy on TME and DNA damage was studied. Biasibetti et al. performed MRI scanning on a C6-Wistar rat GBM model and conducted regression analysis on tumor growth percentage and growth rate and found considerable variation in tumor volume and growth rate among rat models [24]. We scanned the rat skull with MRI and calculated tumor volume on days 21 and 28 of the experimental cycle. It was discovered that the repressive effect of resveratrol and radiation utilized in combination on tumor volume was stronger than that of radiation or resveratrol alone. Moreover, the elevation of body weight and the inhibition of mortality and tumor volume in GBM rats were enhanced by resveratrol, unveiling that resveratrol indeed acted as a radiosensitizer in GBM.

It is reported that the proliferation of malignant tumors is the result of complex regulation participated by multiple factors, among which Ki-67 is the most commonly used and reliable indicator to evaluate the proliferation of malignancies [25]. Our results showed that each intervention group effectively impeded the positive expression of Ki-67, and combined radiation and resveratrol showed stronger suppressive effect. Some researchers clarified that resveratrol could effectively weaken the proliferation and infiltration of GBM cells, and the mechanism was that resveratrol could reduce AKT phosphorylation and induce activation of p53 expression, resulting in the transcription of downstream p53 target genes [26]. Sato et al. pointed out that resveratrol could alleviate the expression of the Nanog gene by activating the p53/p21 signaling pathway in glioma cells [27]. P21 and p53 are key regulators of the transition from the G0/G1 phase to S phase in the cell cycle [28]. In addition, resveratrol can weaken the expression of p-STAT3 and its downstream proteins, such as Survivin, Cyclin D1, COX-2, and C-myc by impeding the phosphorylation activity of STAT3 [29]. STAT3 and its downstream effectors, such as Survivin and Bcl-2, regulate multiple processes that are critical for cancer development, involving tumorigenesis and growth, cell death, cellular senescence, metastasis, and differentiation [30]. Previous report illustrated that GBMs with high expression of Survivin exhibited radioresistance [31]. Some scholars believed that Survivin could restrain the activation of caspase-3, caspase-7, and caspase-9 and weaken apoptosis mediated by them [32–34]. As expected, in the present study, except for the observed promotive effect on cell apoptosis and cell cycle blockage of GBM tumors, we found that radiation, resveratrol, or cotreatment also effectively suppressed Cyclin D1, C-myc, and Survivin levels as well as the ratios of p-STAT3 (ser 727)/STAT3, p-STAT3 (tyr 705)/STAT3, and p-AKT/AKT and upregulated Bad, Cleaved Caspase-9, p-p53, and p-p21 levels in GBM tissues, suggesting that resveratrol triggered apoptosis by downregulating the AKT/STAT3 signaling pathway, thereby enhancing the effect of radiotherapy.

Wang L et al. found that the combination of resveratrol and radiation could induce the differentiation of SU-2 cells, notably intensify the level of autophagy and apoptosis of GBM cells, and decrease the repair of DNA damage caused by radiation [35]. Exposure of cells to radiation can cause a series of DNA damage, while the DNA repair pathway is one of the primary reasons of radiation resistance [36]. Increasingly reports illustrated that combined natural products and radiation could impede DNA repair machinery; thus, some natural products could be radiosensitizing agents [37, 38]. In this study, by conducting comet analysis, DNA damage in the cotreatment groups was more severe relative to that in the single-treated group, which was plausible for understanding its radiosensitization mechanism. Numerous studies illuminated that ATM was a critical protein in DNA self-repair and exhibited a crucial role in DNA double-strand break signaling, thereby phosphorylating H2A.X [39]. The downstream target proteins of ATM can be phosphorylated by the activated ATM, thereby arresting cell growth cycle, preventing DNA repair, and promoting apoptosis [40]. A study reported that *γ*H2A.X was phosphorylated in an ATM-dependent manner in response to DNA damage [41]. By examining the protein expression of ATM and *γ*H2A.X, we uncovered that combined radiation and resveratrol presented stronger promotive effect on the ratios of p-ATM/ATM and p-*γ*H2A.X/*γ*H2A.X in GBM tissues, which involves in the impairment of GBM DNA damage.

Tumor immune microenvironment plays a significant role in the development of tumors. Tumor immunotherapy can restore the surveillance effect of immune cells on tumor cells, preventing immune escape [42]. PD-L1 is expressed on the surface of many tumor cells and related immune cells [43]. Many soluble factors produced by immune cells, such as IFN-*γ*, can act as inducers of PD-L1. Other inflammatory factors, such as TNF-*α*, IL-4, IL-6, TGF-*β*, and IL-10, affect the expression of PD-L1 [44, 45]. The role of resveratrol as tumor immunotherapy has been reported in various tumors [46, 47]. Some scholars stated that resveratrol modulated the changes of Treg population and tumor-related immune cytokines, as reflected by the downregulation of the CD8^+^CD122^+^ Tregs, TGF-*β*1, and IL-10 and the upregulation of IFN-*γ*-expressing CD8^+^T cells, TNF-*α*, and IFN-*γ* in the tumor and peripheral lymphoid organs of tumor-bearing mice [48]. Chen et al. demonstrated that resveratrol intervention effectively weakened Renca tumor growth, and its suppression was dependent on CD8^+^T cells, suggesting that resveratrol could be used in the immunotherapy of renal cell carcinoma by regulating TME [49]. Our study complemented the deficiency of resveratrol in the study of antitumor immunity of the intracranial GBM model. Radiation augmented the percentage of CD3^+^CD8^+^T cells and IFN-*γ*^+^CD8^+^T cells and suppressed the percentage of (CD4^+^CD25^+^Foxp3)/CD4^+^T cells and PD-L1 expression, which was further strengthened by resveratrol. The above results suggested that resveratrol improved GBM immunotherapy by modulating TME.

In conclusion, our results demonstrated that resveratrol elevates the effect of radiotherapy in intracranial GBM tumor. The radiosensitizing effect of resveratrol was supported by arresting cell growth cycle, blocking DNA repair, advancing apoptosis, and inducing antitumor immunity.

## Data Availability

All data generated in this study were included in this article.

## Additional Points

*Highlights*. (i) Resveratrol enhances the inhibition effect of radiation on glioblastoma (GBM) tumor growth and the expression of Ki67 and PD-L1 in an intracranial C6 glioblastoma rat model. (ii) Resveratrol combined with radiotherapy decreases percentage of CD3^+^CD8^+^T and IFN-*γ*^+^CD8^+^T cells and increases percentage of (CD4^+^CD25^+^Foxp3)/CD4^+^T cells in glioblastoma tumor tissues. (iii) Resveratrol combined with radiotherapy blocks the DNA repair and related protein expression in glioblastoma tumor tissues
